# Potential and future strategies for radiotherapy in hepatocellular carcinoma

**DOI:** 10.1111/j.1478-3231.2008.01899.x

**Published:** 2009-02

**Authors:** Daniel M Aebersold

**Affiliations:** Department of Radiation Oncology, Inselspital, Bern University Hospital, University of BernBern, Switzerland

Hepatocellular carcinoma (HCC) continues to be one of the worst cancer conditions worldwide, being the sixth most common cancer and associated with a 5-year overall survival rate of only 5% (1). Surgery, either in terms of local resection or liver transplant, is still the treatment of choice, with 5-year survival rates of 30–70% (2). However, <20% of patients qualify for this treatment because of intrahepatic cancer multifocality, extrahepatic tumour manifestations, inadequate liver function and/or involvement of vascular or biliary structures. Alternative local treatment options for localized HCC include radiofrequency ablation (RFA), percutaneous ethanol injection (PEI), radiotherapy and transarterial chemoembolization (TACE). Two randomized trials have shown that TACE leads to an improved survival compared with best supportive care (3, 4), but its efficacy is modest and restricted to patients without portal vein thrombosis.

The use of radiotherapy for locoregional treatment of HCC has long been studied. The relatively low tolerance of the whole liver to radiotherapy traditionally prohibited the application of sufficiently high doses to control local tumour growth, leading to a general notion of HCC as a ‘radioresistant condition’. However, radiotherapy underwent major technological improvements during the last decades including diagnostic imaging, radiotherapy planning techniques [three-dimensional (3D) conformal radiotherapy, intensity-modulated radiotherapy and stereotactic body radiosurgery], image-guided radiotherapy to detect to the exact position of the tumour at the time of treatment along with respiration-gated radiotherapy to account for liver movements because of breathing. Moreover, radiotherapy with highly conformal protons and carbon ions is increasingly available. All these technological advances allow to treat liver tumours much more precisely, thereby minimizing dose to uninvolved liver tissue and other organs at risk. Because of the opening of the therapeutic window, radiotherapy has lately gained increased consideration for treatment of HCC, specifically in cases of failures to standard treatments [recently reviewed in (5)].

In this issue of *Liver International*, Seong *et al*. (6) report on the practice patterns and outcome in 398 radiation-treated HCC patients in Korea. Data from 10 Korean institutions were collected. The vast majority of patients have been treated by 3D-conformal radiotherapy after failure of TACE. The paper confirms the results of a previous report of the authors on a subset of patients from one single institution, where multivariate analysis revealed increased radiation dose to be an independent prognostic factor for improved overall survival (7). This is also in line with the results of the University of Michigan group, having shown that dose-escalated radiotherapy above 75 Gy is associated with increased overall survival in patients with liver malignancies (8).

In the current paper by Seong *et al*., overall survival was also found to be associated with absence of lymph node involvement and smaller tumour size, the latter being most probably linked to a better local control rate. However, because of the retrospective nature of the current study, no systematic data were available to estimate response rates. In a French prospective phase II trial with 66 Gy given to either single HCCs ≥5 cm or two HCCs≤3 cm, a response rate of 92% has been achieved, the complete response rate being 80% (9). At a median follow-up of 29 months, the in-field local control rate was 78%, indicating the high potential of local tumour cure by radiotherapy.

It is well established that the risk of liver toxicity in radiotherapy-treated patients is both dependent on radiotherapy-related parameters such as dose per irradiated liver volume as well as on host factors including the extent of liver cirrhosis. In the French prospective trial, Child–Pugh class A patients had considerably less liver toxicities (25% grade 3, 0% grade 4) than Child–Pugh class B patients (45% grade 3, 27% grade 4). However, although 3D-conformal radiotherapy planning is widely used, enabling to study partial volumen liver tolerance, systematic data on the interdependence of dose to liver volume, host factors and liver radiotherapy toxicity are scarce. It is strongly recommended to prospectively include such analyses in future radiotherapy trials.

For a long time, systemic chemotherapeutic approaches in HCC have had limited success, with low response rates. Recently, sorafenib – an oral multikinase inhibitor – has been tested successfully in a prospective randomized trial in advanced HCC (10). Given the high rate of incidence of tumour relapses outside the irradiated volumen as well as the radiosensitizing properties of many targeted agents, there is a strong rationale to explore their combination with future high-precision radiotherapy approaches.

## References

[1] Parkin DM, Bray F, Ferlay J (2005). Global cancer statistics, 2002. CA Cancer J Clin.

[2] Bruix J, Sherman M (2005). Management of hepatocellular carcinoma. Hepatology.

[3] Llovet JM, Bruix J (2003). Systematic review of randomized trials for unresectable hepatocellular carcinoma: chemoembolization improves survival. Hepatology.

[4] Lo CM, Ngan H, Tso WK (2002). Randomized controlled trial of transarterial lipiodol chemoembolization for unresectable hepatocellular carcinoma. Hepatology.

[5] Dawson LA (2008). The evolving role of radiation therapy in hepatocellular carcinoma. Cancer Radiother.

[6] Seong J, Lee IJ, Shim SJ (2009). A multicenter retrospective cohort study of practice patterns and clinical outcome on radiotherapy for hepatocellular carcinoma in Korea. Liver Int.

[7] Seong J, Park HC, Han KH (2003). Clinical results and prognostic factors in radiotherapy for unresectable hepatocellular carcinoma: a retrospective study of 158 patients. Int J Radiat Oncol Biol Phys.

[8] Ben-Josef E, Normolle D, Ensminger WD (2005). Phase II trial of high-dose conformal radiation therapy with concurrent hepatic artery floxuridine for unresectable intrahepatic malignancies. J Clin Oncol.

[9] Mornex F, Girard N, Beziat C (2006). Feasibility and efficacy of high-dose three-dimensional-conformal radiotherapy in cirrhotic patients with small-size hepatocellular carcinoma non-eligible for curative therapies–mature results of the French Phase II RTF-1 trial. Int J Radiat Oncol Biol Phys.

[10] Llovet JM, Ricci S, Mazzaferro V (2008). Sorafenib in advanced hepatocellular carcinoma. N Engl J Med.

